# Can we achieve better recruitment by providing better information? Meta-analysis of ‘studies within a trial’ (SWATs) of optimised participant information sheets

**DOI:** 10.1186/s12916-021-02086-2

**Published:** 2021-09-23

**Authors:** Vichithranie W. Madurasinghe, Peter Bower, Sandra Eldridge, David Collier, Jonathan Graffy, Shaun Treweek, Peter Knapp, Adwoa Parker, Jo Rick, Chris Salisbury, Mei See Man, David Torgerson, Rebecca Sheridan, Frank Sullivan, Sarah Cockayne, Charlotte Dack

**Affiliations:** 1 Nuffield Department of Population Health, University of Oxford, Richard Doll Building, Old Road Campus, Roosevelt Drive, Oxford, OX3 7L UK; 2 NIHR School for Primary Care Research, School of Health Sciences, Manchester Academic Health Science Centre, University of Manchester, Manchester, M13 9PL UK; 3 Centre for Clinical Trials and Methodology, Institute of Population Health Sciences, Queen Mary University of London, 58 Turner Street, London, E1 2AB UK; 4 Barts NIHR Biomedical Research Centre, William Harvey Research Institute, Queen Mary University of London, London, EC1M 6BQ UK; 5 Arbury Road Surgery 114, Arbury Road, Cambridge, CB4 2JG UK; 6 Health Services Research Unit, University of Aberdeen, Room 306, 3rd Floor, Health Sciences Building Foresterhill, Aberdeen, AB25 2ZD UK; 7 Department of Health Sciences, University of York & the Hull York Medical School, York, UK; 8 York Trials Unit, Department of Health Sciences, University of York, York, UK; 9 National Institute of Health Research School for Primary Care Research, Manchester Academic Health Science Centre, Centre for Primary Care, University of Manchester, Oxford Road, Manchester, M13 9PL UK; 10 Centre for Academic Primary Care, Department of Population Health Sciences, Bristol Medical School University of Bristol, Canynge Hall, 39 Whatley Road, Bristol, BS8 2PS UK; 11 Norwich Clinical Trials Unit, Norwich Medical School, University of East Anglia, Norwich, NR4 7TJ UK; 12 Department of Health Sciences, University of York, Heslington, York, YO10 5DD UK; 13 University of St. Andrews North Haugh, St. Andrews, Fife, KY16 9T UK; 14 Department of Psychology, University of Bath, Bath, BA2 7AY UK

**Keywords:** Recruitment, Information, User-testing, Research methodology, Randomised controlled trial, SWATs

## Abstract

**Background:**

The information given to people considering taking part in a trial needs to be easy to understand if those people are to become, and then remain, trial participants. However, there is a tension between providing comprehensive information and providing information that is comprehensible. User-testing is one method of developing better participant information, and there is evidence that user-tested information is better at informing participants about key issues relating to trials. However, it is not clear if user-testing also leads to changes in the rates of recruitment in trials, compared to standard trial information. As part of a programme of research, we embedded ‘studies within a trial’ (SWATs) across multiple ongoing trials to see if user-tested materials led to better rates of recruitment.

**Methods:**

Seven ‘host’ trials included a SWAT evaluation and randomised their participants to receive routine information sheets generated by the research teams, or information sheets optimised through user-testing. We collected data on trial recruitment and analysed the results across these trials using random effects meta-analysis, with the primary outcome defined as the proportion of participants randomised in a host trial following an invitation to take part.

**Results:**

Six SWATs *(n*=27,805) provided data on recruitment. Optimised participant information sheets likely result in little or no difference in recruitment rates (7.2% versus 6.8%, pooled odds ratio = 1.03, 95% CI 0.90 to 1.19, *p*-value = 0.63, *I*^2^ = 0%).

**Conclusions:**

Participant information sheets developed through user testing did not improve recruitment rates. The programme of work showed that co-ordinated testing of recruitment strategies using SWATs is feasible and can provide both definitive and timely evidence on the effectiveness of recruitment strategies.

**Trial registration:**

*Healthlines Depression* (ISRCTN14172341)

*Healthlines CVD* (ISRCTN27508731)

CASPER (ISRCTN02202951)

ISDR (ISRCTN87561257)

ECLS (NCT01925625)

REFORM (ISRCTN68240461)

HeLP Diabetes (ISRCTN02123133)

**Supplementary Information:**

The online version contains supplementary material available at 10.1186/s12916-021-02086-2.

## Background

Randomised controlled trials remain the gold standard for evaluating effectiveness of many interventions, but recruitment to trials remains problematic [1, 2]. Regardless of the widespread nature of recruitment challenges in trials, and the negative impacts they have on individual trials, little is known about what recruitment strategies work best with particular participants. One way of testing recruitment strategies is to embed them in real trials using ‘studies within a trial’ (SWATs), where participants in the host trial are randomised to different recruitment methods [3].

A recent Cochrane review of trial recruitment strategies identified 72 strategies but only three with GRADE high certainty evidence, limiting the ability of trial teams to draw on a rigorous evidence base to inform recruitment design [4]. Importantly, many recruitment strategies were the subject of single evaluations, which means that it is difficult to determine whether the effects could be replicated across multiple studies and contexts. The authors concluded that ‘trialists should aim to include evaluations of recruitment strategies in their trials’ (p. 22) [4].

The ‘systematic techniques for assisting recruitment to trials’ (START) research programme funded by the UK Medical Research Council (MRC) responds to these limitations. The START programme was designed to develop the conceptual, methodological and logistical framework to make SWATs a routine part of the delivery of trials, and to assess the feasibility of this approach by developing a small number of recruitment strategies and testing them across multiple host trials in SWATs [5].

In the START programme, we first developed two recruitment strategies: (1) written information optimised through application of information design principles and user-testing (‘participant information sheets optimised through user-testing’) and (2) multimedia information presented via the Internet. We then recruited multiple trials to include a SWAT evaluation of these two recruitment strategies, testing their effectiveness across multiple trials simultaneously [5]. By testing the same strategy across multiple trials, we aimed to provide both a more precise estimate of the effect of the strategy (taking advantage of larger sample size available across multiple trials) and explore the degree to which the effects of recruitment strategies varied across different trial contexts. Moreover, it also offers the opportunity of providing evidence more quickly.

Many individual SWATs within the START programme have now been published [6–10]. In this paper, we synthesise those that evaluated participant information sheets optimised through user-testing.

## Methods

The broad methods underlying the START programme have been published [5].

### Recruitment of host trials

As part of the START programme, chief investigators on trials recently funded by the National Institute of Health Research (NIHR) Health Technology Assessment Programme or on the Primary Care Research Network portfolio were invited to participate in START. Interested trials were selected on the basis of sample size (at least 800 participants to be approached) and design (using a recruitment method amenable to the START recruitment strategies). Although a variety of recruitment methods could be adopted for studies included in the programme (such as postal or face-to-face recruitment), all studies that participated used postal recruitment methods. The minimum sample size of 800 participants to be approached in each trial was based on an indicative sample size calculation, although the expectation was always that the primary analysis would involve pooling of results across trials in a meta-analysis [5]. Host trials were offered access to one of two strategies (participant information sheets optimised through user-testing or multimedia information), both intended to improve communication of trial information to potential participants, which has been shown to have potential to increase research participation rates [11]. We aimed to recruit 6 ‘host’ trials to each strategy. This was based on practical considerations and a desire to test the strategy in a reasonable range of contexts rather than a formal sample size calculation.

### Development of the intervention—participant information sheets optimised through user-testing

For user-testing, we recruited healthy members of the public, who had a similar socio-demographic profile (age and education) to the participants eligible for host trials. We excluded people who had taken part in any medicines trial or readability testing in the previous 6 months.

An independent groups design was used, with each participant seeing only one version of the information. We conducted three rounds of user-testing, with 10 participants in each round. The first round tested the original trial materials (PIS and cover letter), after which the optimised versions were developed, using information design and plain English. Although the information sheet and letter for each trial varied the revisions always included: plain English; short sentences and paragraphs; use of colour for contrast and impact, and bold text for highlighting; a reduced number of sub-sections; a contents list; and clear trial contact details. This approach has been shown to produce increased levels of understanding and approval [12–14].

The second and third rounds tested the revised versions, with minor changes made to wording and layout in response to the findings of each round of testing. In user-testing, each participant was shown a version of the information sheet and cover letter and asked to respond to 20 factual questions: three related to the cover letter and 17 to the information sheet. The questions were drawn from four categories of information that would apply to any trial: the nature and purpose of the trial (three questions); the process and meaning of consent (four questions); trial procedures (10 questions); and safety, efficacy and nature of the tested intervention (three questions). For each question, participants were asked to locate the answer (testing navigation and organisation of the information), then give the answer in their own words (testing clarity of wording) [15].

### Methods of the SWAT

In each SWAT, participants being approached to take part were randomised to receive the optimised information or routine information materials. Individual randomisation was preferred for the SWATs, as the methods used were highly amenable to randomisation at that level, which would generally increase power and precision, and be less vulnerable to selection bias. We adopted site randomisation only where that was preferred for logistical reasons (e.g. where there was insufficient resource to conduct individual randomisation, or where individual randomisation might cause disruption to the host trial).

### Outcome measures

The primary outcome was recruitment, defined as the proportion of participants recruited and randomised to a host trial following an invitation to take part. The denominator for the outcome was the total number of potentially eligible participants offered entry to the host trial. Depending on the particular trial, this would include a mix of eligible and ineligible patients according to the formal inclusion and exclusion criteria. All trials were able to provide reliable data on the numbers offered participation.

Secondary outcomes were:

- Acceptance, defined as the proportion of potentially eligible participants who express interest in participating, either by posting a reply or attending a recruitment appointment. We anticipated that in some SWATs, the number of participants recruited to the host trial could be different from numbers of participants responding positively to the invitation, due to eligibility criteria used in the host trial.

- Decline, defined as the proportion of participants who actively decline to participate in the host trial.

### Research ethics approval

The START programme was approved by the National Research Ethics Service (NRES) Committee, Yorkshire and the Humber – South Yorkshire (Ref: 11/YH/0271) on 5 August 2011. Each individual host trial had its own ethical agreement and registration.

### Data analysis

For each individual SWAT, analyses of recruitment were conducted in line with the statistical analysis plan developed by SE and VM. Outcomes were first described separately by study arm and then compared using logistic regression to estimate the between-group odds ratio and corresponding 95% confidence interval.

For the pooled analysis, data from each SWAT were entered into Stata and meta-analysed using the Stata *metan* command (Stata version 14.2). Random effects meta-analysis models were used based on the assumption that clinical and methodological heterogeneity was likely to impact on the results. Statistical inconsistency was quantified using the *I*^2^ statistic.

In the meta-analysis, we used a two-staged analysis strategy where each individual SWAT was analysed using the appropriate analysis methods (i.e. taking into account whether it was individually randomised or cluster randomised) to generate trial-level summary statistics (e.g. odds ratio) first, and then the results from each individual SWATs were combined across trials using the Stata metan command (Stata version 14.2).

Regardless of the observed statistical heterogeneity, we performed pre-specified subgroup analyses investigating differences between studies based on underlying recruitment rates (low defined as a recruitment rate of 5% or below in control group vs. higher rates). We hypothesised that when the baseline recruitment rate is low, the increase in the absolute recruitment rate associated with a recruitment intervention is likely to be higher. A second planned analysis comparing patients with a known diagnosis versus participants ‘at risk’ was not conducted as it proved difficult to assign trials to the categories reliably. In a post hoc sensitivity analysis, we assessed the impact of including one of the SWATs (ISDR) which faced particular design challenges [9] on the overall pooled effect estimate by re-estimating the pooled odds ratio with this study excluded.

## Results

We originally recruited 8 host trials to the START programme. Although it proved difficult to record exactly how many host trial teams were approached, as teams became aware of the programme through a variety of means including presentations and word of mouth, we estimated that at least 225 were contacted. One of the eight host trials was unable to deliver data, as the trial finished before the necessary permissions were in place to do the SWAT. We therefore conducted SWATs in 7 host trials (Table 1). One SWAT only reported participant expressions of interest and not trial recruitment. All but one of the SWATs has been published, and we report the unpublished SWAT (Additional file 1 Table S1) according to current guidelines [16].

**Table 1:** Trial characteristics

Trial name	Population	Host trial intervention and comparison	Design of the host trial	Design of SWAT
CASPER [10]	Patients at least 65 years of age with sub-threshold depression	Intervention: collaborative care including screening for depression, collaborative care and low intensity psychological intervention plus usual GP care Comparator: usual GP care alone	Individually randomised two-arm parallel group trial	Individually randomised two-arm parallel group trial
ECLS [8]	Current or ex-smokers aged 50 to 75 years	Intervention: A new blood test named EarlyCDT-Lung test to detect seven autoantibodies to aid in the risk assessment and early detection of lung cancer Comparator: standard care	Individually randomised two-arm parallel group trial	Individually randomised two-arm parallel group trial
Healthlines CVD [7]	Those aged 40 to 74 years with increased risk of cardiovascular disease	Intervention: NHS Direct-delivered telehealth intervention including telephone support and computer-based self-management to support patients Comparator: usual care	Individually randomised two-arm parallel group trial	Individually randomised two-arm parallel group trial
Healthlines Depression [7]	Those at least 18 years of age and having a confirmed diagnosis of clinical depression	Intervention: NHS Direct-delivered telehealth intervention including telephone support and computer-based self-management to support patients Comparator: usual care	Individually randomised two-arm parallel group trial	Individually randomised two-arm parallel group trial
HeLP Diabetes [unpublished]	Adults aged 18 or over, with type 2 diabetes	Intervention: facilitated and supported access (1—an introductory session with nurse introducing HeLP Diabetes web-based programme, 2—supportive follow-up phone calls, and 3—on-going discussion of patient’s self-management goals in routine appointments for diabetes-related matters) to healthcare for patients with diabetes) Comparator: an introductory session with nurse and follow-up phone calls	Individually randomised two-arm parallel group trial	Two-arm randomised trial, clustered by general practice
ISDR [9]	People with diabetes undergoing annual screening for diabetic retinopathy	Intervention: personalised risk based screening intervals Comparator: annual screening	Individually randomised two-arm parallel group trial	Two-arm randomised trial, clustered by week of recruitment
REFORM [6]	Podiatry patients over the age of 65 years	Intervention: multifaceted foot and lower limb intervention for prevention of falls Comparator: a leaflet with fall prevention advice plus continue with current podiatry treatment	Two-arm open randomised cohort controlled trial	Individually randomised three-arm cohort trial

Table 1 describes the characteristics of the seven SWATs (*n*=28,476). Five SWATs randomised participants at the individual level, whereas 2 randomised by cluster (general practice or week of recruitment) because this was operationally easier. All host trials were individually randomised. There were a mix of host trials in adults with physical or mental health conditions, testing a variety of screening and treatment interventions conducted in primary health care settings.

**Fig. 1:**
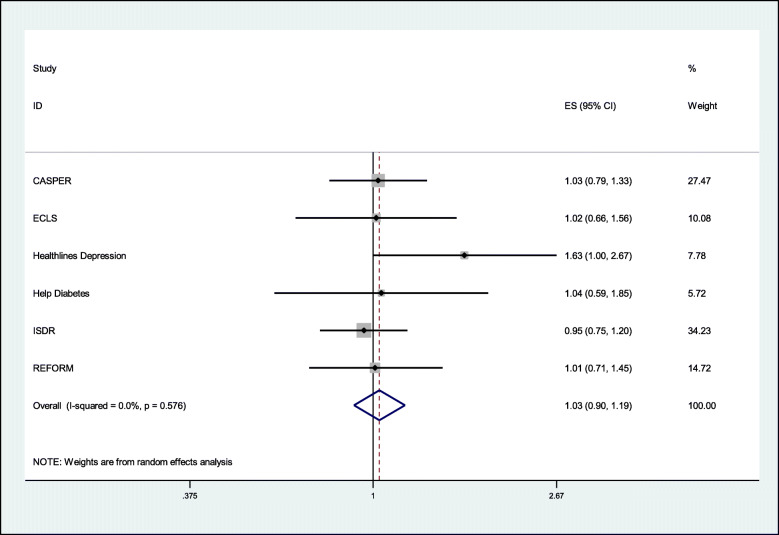
Meta-analysis of primary outcome—randomisations to the host trial

Six SWATs (*n*=27,805) provided data on recruitment. Optimised information likely results in little or no difference in recruitment rates (pooled odds ratio = 1.03, 95% CI 0.90 to 1.19, *p*-value = 0.630, *I*^2^ = 0%) (Table 2 and Fig. 1).

**Table 2:** Primary outcome - randomised to host trial

Study	Optimised	Standard	Odds ratio	95% CI	% weight
CASPER	116 / 5765	113 / 5766	1.027	0.791 to 1.334	27.5
ECLS	180 / 1136	176 / 1126	1.016	0.660 to 1.564	10.1
Healthlines (Depression)	43 / 682	27 / 682	1.630	1.000 to 2.670	7.8
Help Diabetes	183 / 2510	166 / 2370	1.044	0.589 to 1.852	5.7
ISDR	422 / 1666	393 / 1503	0.951	0.752 to 1.201	34.2
REFORM	63 / 2301	62 / 2298	1.010	0.710 to 1.450	14.7
Pooled	**1007 / 14,060 (7.2%)**	**937 / 13,745 (6.8%)**	**1.034**	**0.902 to 1.186**	**100.0**

Heterogeneity chi-squared = 3.82, *p* = 0.576; *I*^2^ = 0.0%; estimate of between-study variance = 0.000; test of pooled odds ratio = 1: *z* = 0.48, *p* = 0.630

Sensitivity analysis excluding ISDR trial results: pooled odds ratio = 1.08 (95% CI 0.913 to 1.279, *p* = 0.370); *I*^2^ = 0.0%, *p* = 0.547

Optimised participant information sheets were not differentially effective in trials with different baseline recruitment rates: trials with low rates (pooled odds ratio 1.12, 95% CI 0.88 to 1.44, *p*-value = 0.363, 3 trials, *n*=17,494) vs high rates (pooled odds ratio 0.97, 95% CI 0.80 to 1.18, *p*-value = 0.790, 3 trials, *n*=10,311).

Seven SWATs (*n*=28,476) provided data on participant acceptance rates. Participants receiving optimised participant information sheets were not more likely to respond positively to the invitation compared to participants receiving standard information (pooled odds ratio 1.06, 95% CI 0.99 to 1.14, *p* value = 0.098, *I*^2^ = 0%).

Three SWATs (*Healthlines Depression, Healthlines CVD* and *CASPER*, *n*=13,566) provided data on decline rates. Participants receiving optimised participant information sheets showed little or no difference in rates of declining than those receiving standard participant information sheets (pooled odds ratio was 1.03, 95% CI 0.96 to 1.10, *p* value = 0.446, *I*^2^ = 0%, where a higher odds ratio meant they were more likely to decline).

There was little or no statistical heterogeneity across studies on all our comparisons, and the sensitivity analyses excluding ISDR study did not change our meta-analysis findings.

## Discussion

### Summary

Participant information sheets optimised through information design and user-testing did not improve recruitment rates. There was also no evidence that the intervention had any wider impact on participant behaviour, either in agreeing to participate in principle (prior to eligibility assessment) or actively declining the trial.

The programme of work showed that co-ordinated testing of recruitment strategies using SWATs is feasible and can provide definitive evidence on the effectiveness of recruitment strategies.

### Limitations of the study

The host trials undertaking the SWATs were self-selected, and therefore, the studies on which the programme was run represent a relatively specific group of study contexts. It is likely that the variation in those contexts was sufficient to give the recruitment strategy a fair test across multiple designs and populations, and there was limited evidence of significant variation in effect. All the included trials used postal recruitment to invite participants.

The START programme was co-ordinated and represents the most concentrated evidence synthesis in the area of recruitment to date, according to the latest Cochrane review [4]. Nevertheless, even the pooled analysis of data from 6 trials left some imprecision in the estimate of effect. The creation and dissemination of the evidence was far from rapid, given recruitment began in 2012. This reflects a number of issues, including the fact that some SWATs extended beyond the funded START programme itself (hampering completion of the meta-analysis). Some individual SWATs were slow to complete recruitment or provide recruitment data, and the summary meta-analysis was reported only after all SWATs had the opportunity to be published individually. Development of SWAT processes since that time has highlighted the need for greater efficiency, permitting faster publication of individual studies and ‘living’ meta-analyses at the level of a strategy to better inform the trials community. The participating trials were led by experienced investigators and teams, so the standard information sheets used in the control arm may have already been well designed based on their experience of recruitment challenges in previous trials, leaving less scope for improvement through user-testing. All the host trials were done in the UK, making it unclear how applicable this evidence is to other countries.

### Study results in the context of the wider literature

We report here a linked series of pre-planned and co-ordinated SWATs testing the same recruitment intervention, rather than a retrospective systematic review of all relevant studies using this strategy. The studies reported here will eventually be integrated into the ongoing Cochrane review on strategies to improve trial recruitment [4], alongside similar data from studies outside the START programme.

After we began our programme of research, guidance was published to help trial teams and funders decide if another evaluation of a SWAT intervention was required [17]. We present the guidance and our judgements based on our meta-analysis (Additional file 2: Table S2 and Additional file 2: Figure S1). Overall, the current evidence would suggest that most criteria are no longer met and that further SWAT evaluations of this strategy would not be a high priority in the UK.

### Implications for policy makers and researchers

Our data suggest that although optimising information though user-testing leads to improved comprehension, it is not likely to translate to improved trial recruitment. Our programme of work did show that co-ordinated testing of recruitment strategies using SWATs is feasible. SWATs done across multiple host trials provide a body of evidence to help trialists to make their trial process decisions more evidence-informed, which is far from routine at present because there are little data for trialists to use. There are several methods of supporting further SWAT studies. One is further bespoke funding similar to START, for example the TRECA study [18] which replicates START in the context of multimedia decision aids for children and adolescents. The MRC-funded PROMETHEUS study was funded to extend the START model with a larger number of trials and for strategies targeting recruitment or retention.

Another model has been to build SWATs into host trials as part of the planning for the host trial itself. For example, the UK’s NIHR Health Technology Assessment funding scheme offers trials additional money to embed SWATs as part of the funding bid. Ireland’s Health Research Board (HRB) has a bespoke SWAT funding scheme run through the HRB-Trial Methodology Research Network. Each model of delivery has advantages and disadvantages but the key issue is that SWATs, while generally cheap, are not free, and therefore need funding streams that support them.

## Conclusions

Although we have shown the feasibility of a co-ordinated programme of SWATs among multiple trials, the challenge is to expand and accelerate the process in order to build the evidence base more rapidly and fully.

However, there are two additional priorities beyond increases in the volume and efficiency of SWATs. First, it is critical that any SWAT programme can clearly identify strategies that can be implemented (or de-implememented) and is able to ensure that findings regarding the effect of these strategies are rapidly disseminated to trials units and research teams to change practice in line with the developing evidence base.

Secondly, and more importantly, it will be critical to show that these efforts lead to better trials—which might include more efficient delivery (quicker approvals process or quicker recruitment at lower cost), more participant-centred trials (better aligned to participant needs and providing better participant experience, which may improve trial retention) and recruitment of more diverse and representative populations.

## Supplementary Information

**Additional file 1: Table S1.** Details of the unpublished Help Diabetes SWAT

**Additional file 2: Table S2.** Criteria for deciding whether user tested participant information needs another SWAT evaluation. **Figure S1.** Cumulative meta-analysis

## Data Availability

The summary meta-analysis data is available from the corresponding author.

## Abbreviations

MRC: Medical Research Council
NRES: National Research Ethics Service
PIS(s): Participant information sheet(s)
REC: Research Ethics Committee
START: Systematic Techniques for Assisting Recruitment to Trials
SWAT: Study within a trial
