# Supplementary material for: Can we achieve better recruitment by providing better information? Meta-analysis of ‘studies within a trial’ (SWATs) of optimised participant information sheets
Source: BMC Med. 2021 Sep 23;19:218. doi: 10.1186/s12916-021-02086-2 (PMC8459527; doi:10.1186/s12916-021-02086-2)
Supplement: Supplementary file 1 — Additional file 1: Table S1. Details of the unpublished Help Diabetes SWAT [file 12916_2021_2086_MOESM1_ESM.docx]

**Additional file 1 Table S1 Details of the unpublished Help Diabetes SWAT**

| **Section/topic and item no.** | **CONSORT 2010 (standard) checklist item** | **Extension for embedded recruitment trials** |  |  |
| --- | --- | --- | --- | --- |
| Title and abstract |  |  |  |  |
| 1a | Identification as a randomised trial in the title | Identification as an *embedded randomised recruitment trial* in the title | Effects of a Patient Information Sheet optimised through user testing on the number of patients recruited in the HeLP-Diabetes randomised controlled trial: a study within a trial (SWAT) |  |
| 1b | Structured summary of trial design, methods, results, and conclusions (for specific guidance see CONSORT for abstracts) | Structured summary of *embedded recruitment trial* design, methods, results, and conclusions (for specific guidance see CONSORT for abstracts) | As the embedded study is unpublished, this appendix represents the structured summary |  |
|  | Introduction | | | |
|  | Background and objectives | | | |
| 2a | Scientific background and explanation of rationale | Scientific background and explanation of rationale for the *embedded recruitment trial including a brief description of the host trial(s) as appropriate* | Recruitment into clinical trials is a common challenge experienced by researchers. Patient Information Sheets (PIS) are used to provide patients with information about a trial. This has to be detailed enough in order for a patient to make an informed decision about whether they would like to take part or not. This means PIS are often long, complex and visually unappealing written documents following a standard template which may have a negative impact on recruitment rates. One method that has been shown to improve patients understanding of this information is user testing. |  |
| 2b | Specific objectives or hypotheses | Specific objectives or hypotheses for the *embedded recruitment trial* | The SWAT was done as an embedded randomised trial in the NIHR funded HeLP-Diabetes study as part of the MRC START initiative to evaluate whether optimising PIS through user testing improves trial recruitment. |  |
|  | Methods | | | |
|  | Trial design | | | |
| 3a | Description of trial design (such as parallel, factorial) including allocation ratio | Description of *embedded recruitment trial* design (such as parallel, factorial, *cluster*) including allocation ratio | The SWAT was a two-arm randomised trial, clustered by general practice (allocation ratio 1:1) |  |
| 3b | Important changes to methods after trial commencement (such as eligibility criteria), with reasons | Important changes to methods of the *embedded recruitment trial* after commencement (such as eligibility criteria), with reasons | None |  |
|  | Participants | | | |
| 4a | Eligibility criteria for participants | Eligibility criteria for participants for the *embedded recruitment trial, including any differences from those for the host trial(s)* | Practices were required to have two nurses – one to deliver and facilitate the intervention, and one to collect data. Participants eligible for inclusion in the host HeLP-Diabetes trial were adults, aged 18 or over, with type 2 diabetes, registered with participating general practices.  Patients were excluded if they were unable to provide informed consent; unable to use a computer due to severe mental or physical impairment; had insufficient spoken or written English to use the intervention (operationalised as unable to consult without an interpreter); were terminally ill with less than 12 months life expectancy; or were currently participating in a trial of an alternative self-management programme.  There were no additional eligibility criteria for the SWAT. |  |
| 4b | Settings and locations where the data were collected | Settings and locations where the *embedded recruitment trial was carried out, including a brief description of the host trial(s) as appropriate* | 21 general practices in England were recruited to the HeLP-Diabetes trial. 2 of these were pilot practices recruited before the start of the embedded trial. Therefore a total of 19 general practices in England were recruited into the embedded trial |  |
|  | Interventions | | | |
| 5 | The interventions for each group with sufficient details to allow replication, including how and when they were actually administered | The interventions for each group *(including control group) within the embedded recruitment trial* with sufficient details to allow replication, including how, where and when they were actually administered | Once recruited, general practices were randomly allocated by the trial manager to either the control arm or the intervention arm of the SWAT.  Practices in the control arm received the original PIS, based on the NHS ethics template and practices in the intervention arm received the optimised, user tested PIS.  Practices then mailed all eligible patients at their practice their allocated PIS inviting them to take part in the HeLP-Diabetes Trial. |  |
|  | Outcomes | | | |
| 6a | Completely defined pre-specified primary and secondary outcome measures, including how and when they were assessed | Completely defined pre-specified primary and secondary outcome measures for the *embedded recruitment trial,* including how and when they were assessed | The primary outcome was the difference in the proportion of patients randomised to the HeLP-Diabetes Trial in the intervention and control group respectively following an invitation to take part. |  |
| 6b | Any changes to trial outcomes after the trial commenced, with reasons | Any changes to *embedded recruitment* trial outcomes after the *embedded recruitment trial* commenced, with reasons | None |  |
|  | Sample size | | | |
| 7a | How sample size was determined | How sample size for the *embedded recruitment trial* was determined | The target sample size of the HeLP-Diabetes host trial was 350 participants.  There was no formal power calculation conducted to determine the SWAT sample size, given that the sample size was restricted by the numbers approached in the HeLP-Diabetes host trial.  However, a minimum sample size of 400 patients to be approached (not randomised) was required to take part in START |  |
| 7b | When applicable, explanation of any interim analyses and stopping guidelines | When applicable, explanation of any interim analyses and stopping guidelines for the *embedded recruitment trial* | None |  |
|  | Randomisation | | | |
|  | Sequence generation | | | |
| 8a | Method used to generate the random allocation sequence | Method used to generate the random allocation sequence for the *embedded recruitment trial* | Each time a general practice was recruited to the trial, the statistician emailed the trial manager the practice allocation (e.g., intervention or control PIS). The trial manager then provided the practice (either by post or in person) with either the intervention PIS or control PIS for the practice nurse to send out to all eligible patients at the practice. |  |
| 8b | Type of randomisation; details of any restriction (such as blocking and block size) | Type of randomisation; details of any restriction (such as blocking and block size) in the *embedded recruitment trial* | Randomisation was conducted in a 1:1 ratio without stratification, at the general practice level |  |
|  | Allocation concealment mechanism | | | |
| 9 | Mechanism used to implement the random allocation sequence (such as sequentially numbered containers), describing any steps taken to conceal the sequence until interventions were assigned | Mechanism used in the *embedded recruitment trial* to implement the random allocation sequence (such as sequentially numbered containers), describing any steps taken to conceal the sequence until interventions were assigned | Computer-generated randomised sequence. |  |
|  | Implementation | | | |
| 10 | Who generated the random allocation sequence, who enrolled participants, and who assigned participants to interventions? | Who generated the random allocation sequence(s), who enrolled participants, and who assigned participants to *embedded recruitment* interventions? | The statistician involved in the HeLP-Diabetes trial |  |
|  | Blinding | | | |
| 11a | If done, who was blinded after assignment to interventions (for example, participants, care providers, those assessing outcomes) and how? | If done, who was blinded after assignment to *embedded recruitment* interventions (for example, participants, care providers, those assessing outcomes) and how? | Both practices and patients were blinded to their allocation |  |
| 11b | If relevant, description of the similarity of interventions | If relevant, description of the similarity of interventions in the *embedded recruitment trial* | Not applicable |  |
|  | Statistical methods | | | |
| 12a | Statistical methods used to compare groups for primary and secondary outcomes | Statistical methods used to compare groups for primary and secondary outcomes of the *embedded recruitment trial* | The data were analysed as part of the START meta-analysis by the START statisitcian (VM) |  |
| 12b | Methods for additional analyses, such as subgroup analyses and adjusted analyses | Methods for additional analyses, such as subgroup analyses and adjusted analyses for the *embedded recruitment trial* | Additional analyses conducted as part of the START meta analysis are described in the main paper |  |
|  | Results | | | |
|  | Participant flow (a diagram is strongly recommended) | | | |
| 13a | For each group, the numbers of participants who were randomly assigned, received intended treatment, and were analysed for the primary outcome | For each group in the *embedded recruitment trial,* the numbers of participants who were randomly assigned, received intended treatment, and were analysed for the primary outcome | See flow diagram |  |
| 13b | For each group, losses and exclusions after randomisation, together with reasons | For each group, losses and exclusions after randomisation to the *embedded recruitment trial,* together with reasons | See flow diagram |  |
|  | Recruitment | | | |
| 14a | Dates defining the periods of recruitment and follow-up | Dates defining the periods of recruitment and follow-up *for both embedded recruitment trial and host trial(s)* | Recruitment for the host trial HeLP-Diabetes took place between September 2013 and December 2014. Follow up data was collected within 10-14 months of randomisation (July 2014 to February 2015) |  |
| 14b | Why the trial ended or was stopped | Why the *embedded recruitment trial* ended or was stopped | Not applicable |  |
|  | Baseline data | | | |
| 15 | A table showing baseline demographic and clinical characteristics for each group | *If possible* a table showing baseline characteristics *of each arm of the embedded recruitment trial* |  |  |
|  | Numbers analysed | | | |
| 16 | For each group, number of participants (denominator) included in each analysis and whether the analysis was by original assigned groups | For each group in the *embedded recruitment trial,* number of participants (denominator) included in each analysis and whether the analysis was by original assigned groups | The data are presented in the main paper. |  |
|  | Outcomes and estimation | | | |
| 17a | For each primary and secondary outcome, results for each group, and the estimated effect size and its precision (such as 95 % confidence interval) | For each primary and secondary outcome, results for each group in the *embedded recruitment trial,* and the estimated effect size and its precision (such as 95 % confidence interval) | The data are presented in the main paper. |  |
| 17b | For binary outcomes, presentation of both absolute and relative effect sizes is recommended | For binary outcomes in the *embedded recruitment trial*, presentation of both absolute and relative effect sizes is recommended | The data are presented in the main paper. |  |
|  | Ancillary analyses | | | |
| 18 | Results of any other analyses performed, including subgroup analyses and adjusted analyses, distinguishing pre-specified from exploratory | Results of any other analyses performed for the *embedded recruitment trial*, including subgroup analyses and adjusted analyses, distinguishing pre-specified from exploratory | The data are presented in the main paper. |  |
|  | Harms | | | |
| 19 | All important harms or unintended effects in each group (for specific guidance see CONSORT for harms) | All important harms or unintended effects in each group *for both the embedded recruitment trial and host trial(s)* (for specific guidance see CONSORT for harms) | No harms were measured, although it was plausible that the user tested PIS could reduce recruitment |  |
|  | Discussion | | | |
|  | Limitations | | | |
| 20 | Trial limitations, addressing sources of potential bias, imprecision, and, if relevant, multiplicity of analyses | *Embedded recruitment trial* limitations, addressing sources of potential bias, imprecision, and, if relevant, multiplicity of analyses | The generalisability of the SWAT was limited by the specific nature of the host trial, but the data were analysed as part of the START meta-analysis to enhance generalisability. |  |
|  | Generalisability | | | |
| 21 | Generalisability (external validity, applicability) of the trial findings | Generalisability (external validity, applicability) *of the embedded recruitment trial* findings | The generalisability of the SWAT was limited by the specific nature of the host trial, but the data were analysed as part of the START meta-analysis to enhance generalisability. |  |
|  | Interpretation | | | |
| 22 | Interpretation consistent with results, balancing benefits and harms, and considering other relevant evidence | Interpretation consistent with results *of the embedded recruitment trial,* balancing benefits and harms, and considering other relevant evidence | The data were analysed as part of the START meta-analysis by the START statisitician (VM). |  |
|  | Other information | | | |
|  | Registration | | | |
| 23 | Registration number and name of trial registry | Registration number and name of trial registry *(for all host trials and embedded recruitment trial if available)* | Registration details for MRC START are in the main paper.  HeLP-Diabetes ISRCTN02123133 |  |
|  | Protocol | | | |
| 24 | Where the full trial protocol can be accessed, if available | Where the *embedded recruitment trial* protocol can be accessed, if available | No protocol is available |  |
|  | Funding | | | |
| 25 | Sources of funding and other support (such as supply of drugs), role of funders | For the *embedded recruitment trial,* sources of funding and other support, role of funders *and collaborators* | Funding details for MRC START are in the main paper. |  |

19 practices

156 retained at 3 months

156 retained at 12 months

265 patients showing an interest in participating

166 patients randomised

2370 invitations with usual PIS

9 control practices

168 retained at 3 months

164 retained at 12 months

362 patients showing an interest in participating

183 patients randomised

2510 invitations with user tested PIS

10 intervention practices
