# Supplementary material for: Can we achieve better recruitment by providing better information? Meta-analysis of ‘studies within a trial’ (SWATs) of optimised participant information sheets
Source: BMC Med. 2021 Sep 23;19:218. doi: 10.1186/s12916-021-02086-2 (PMC8459527; doi:10.1186/s12916-021-02086-2)

**Additional File 2 Table S2 Criteria for deciding whether user tested participant information needs another SWAT evaluation**

| Criterion | Comment | Criterion met? |
| --- | --- | --- |
| 1. GRADE: the GRADE [22] certainty in the evidence for all key outcomes is lower than ‘high’ | GRADE = moderate  (Downgraded one level for inconsistency) | Yes |
| 2. Cumulated evidence: the cumulative meta-analysis shows that the effect estimate for each outcome essential to make an informed decision has not converged | Although there was some earlier inconsistency, the effect estimate does look to have converged on risk difference of around 0.1% (see additional file Figure 3) | No |
| 3. Context: the range of host trial contexts evaluated to date does not translate easily to the context of the proposed SWAT.d For the proposed SWAT consider PICOT [23]: • P – is the population in the host trial so different from those already included that the current evidence does not provide sufficient certainty? • I – are the health interventions in the host trial so different from those already included that the current evidence does not provide sufficient certainty? • C – is the comparator in the host trial so different from those already included that the current evidence does not provide sufficient certainty? • O – is the SWAT outcome(s) so different to those used in the existing evaluations that that the current evidence does not provide sufficient certainty? • T – in the time since the existing evaluations were done, have regulatory, technological or societal changes made those evaluations less relevant? | All trials were done in the UK. Men and women were involved from age 18 upwards. All six trials were non-drug trials. Two trials targeted depression; one lung cancer; one cardiovascular disease; two diabetes. One depression trial, the lung cancer trial and one diabetes trial were screening trials; the others were self-management of chronic disease. All trials were wholly or partially (ISDR). primary care/community-based. Standard care was the comparator for all trials.  There are a range of contexts put some important gaps, particularly around hospital-based care, drug trials and trials done outside the UK. | Partially met. |
| 4. Balance – participants: the balance of benefit and disadvantage to participants in the host trial and/or the SWAT is not clear | Previous evidence suggests that the optimised format improves understanding, which would be the main benefit for participants. Harms are unclear but likely to be very small, if any. There was no evidence of harm from the six SWAT evaluations. | No |
| 5. Balance – host trial: the balance of benefit and disadvantage to the new host trial is not clear | The main potential benefit to the host trial is increased recruitment and perhaps retention. The impact on the former is largely known in a UK context; retention is unclear. The main disadvantage is cost but this is quantifiable and in the region of £5000 - £8000. | No |

**Additional file 2 Figure S1 Cumulative meta-analysis**


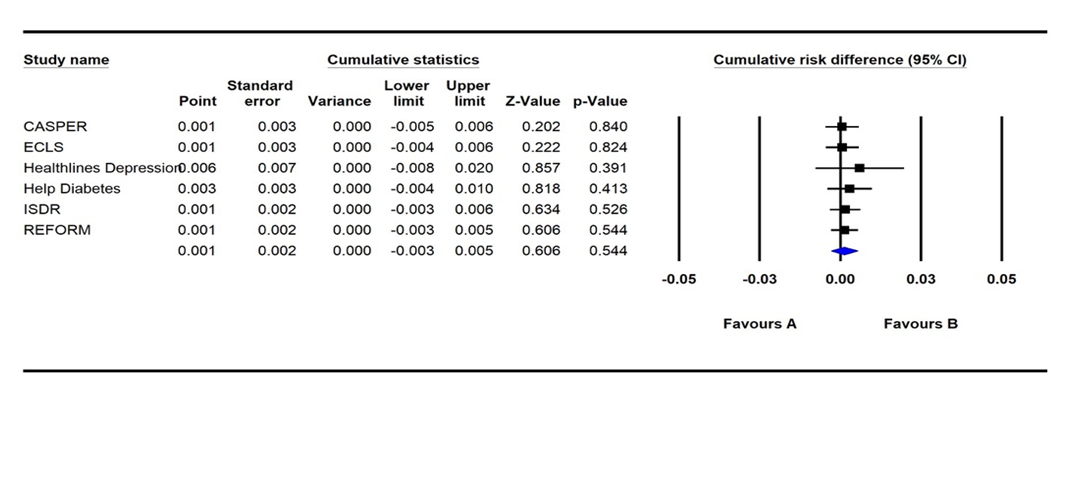

Supplement: Supplementary file 2 — Additional file 2: Table S2. Criteria for deciding whether user tested participant information needs another SWAT evaluation. Figure S1. Cumulative meta-analysis [file 12916_2021_2086_MOESM2_ESM.docx]
